# Evaluation of a telemedicine-based training for final-year medical students including simulated patient consultations, documentation, and case presentation

**DOI:** 10.3205/zma001387

**Published:** 2020-12-03

**Authors:** Sigrid Harendza, Julia Gärtner, Elena Zelesniack, Sarah Prediger

**Affiliations:** 1 Universitätsklinikum Hamburg-Eppendorf, III. Medizinische Klinik, Hamburg, Germany

**Keywords:** assessment, communication, competence, consultation, simulation, telemedicine, training

## Abstract

**Background:** Focused history taking, knowledge-based clinical reasoning, and adequate case presentation during hand-offs represent important facets of competence of practicing physicians. Based on a validated 360-degree assessment simulating a first day of residency we developed a training for final-year medical students including patient consultation, patient management, and patient hand-off. Due to the COVID-19 pandemic the training was changed to a telemedicine format and evaluated.

**Methods: **In 2019, 103 final-year students participated in a newly designed competence-based training including a consultation hour with simulated patients, a patient management phase with an electronic patient chart, and a case presentation in hand-off format. Due to social distancing regulations, the training was not allowed to take place in this way. Therefore, we changed the training to a telemedicine format. In May 2020, 32 students participated in the telemedicine training. A 5-point Likert scale (1: does not apply to 5: fully applies) was used for the evaluation items. The two formats were compared with t-tests.

**Results: **The students were similarly satisfied with the content of the training independently of its format. Both groups found the patient cases interesting (presence: 4.68 ± 0.49, telemedicine: 4.66 ± 0.48). With respect to the telemedicine format, participants were glad that an option had been found that could be offered throughout the final year (4.94 ± 0.24) despite the COVID-19 pandemic and they regarded it as a very useful training for their final examination (4.94 ± 0.24).

**Conclusion: **The telemedicine format of the competence-based training worked as well as the presence format. In its telemedicine format, the training can be offered to students independently of their location.

## Zusammenfassung

**Hintergrund: **Fokussierte Anamneseerhebung, wissensbasiertes klinisches Argumentieren und adäquate Fallpräsentationen in Übergabegesprächen stellen wichtige Kompetenzfacetten praktizierender Ärzt*innen dar. Basierend auf einer validierten 360-Grad Beurteilung eines simulierten ersten Arbeitstags in der ärztlichen Weiterbildung im Krankenhaus entwickelten wir ein Training für Studierende im Praktischen Jahr (PJ) mit einer Sprechstunde, einer Patient*innenbetreuungsphase und einem Übergabegespräch. Aufgrund der COVID-19 Pandemie wurde das Training in ein telemedizinisches Format umgewandelt und evaluiert.

**Methoden:** Im Jahr 2019 nahmen 103 PJ-Studierende an einem neu entwickelten, kompetenzbasierten Training teil, das seine Sprechstunde mit Simulationspatient*innen, eine Patient*innenbetreuungsphase mit einer elektronischen Patient*innenakte und eine Patient*innenfallpräsentation im Übergabeformat beinhaltete. Aufgrund der Abstandsregeln gab es keine Genehmigung, das Training in dieser Weise durchzuführen. Daher wandelten wir das Training in ein Telemedizinformat um. Im Mai 2020 nahmen 32 Studierende an dem Telemedizintraining teil. Eine 5-Punkte Likert Skala (1: trifft nicht zu, bis 5: trifft voll zu) wurde für die Evaluationsitems eingesetzt. Die beiden Formate wurden mittels t-Test verglichen.

**Ergebnisse: **Die Studierenden waren unabhängig vom Trainingsformat gleichermaßen mit den Inhalten des Trainings zufrieden. Beiden Gruppen fanden die Patient*innenfälle interessant (Präsenz: 4,68±0,49; Telemedizin: 4,66±0,48). In Bezug auf das Telemedizinformat waren die Teilnehmenden froh, dass eine Möglichkeit gefunden wurde, die trotz der COVID-19 Pandemie über das gesamte PJ hinweg durchgeführt werden kann (4,94±0,24) und sie erachteten das Format als sehr nützliches Training für ihre Abschlussprüfung (4,94±0,24).

**Schlussfolgerung: **Das Telemedizinformat des kompetenzbasierten Trainings funktionierte genauso gut wie das Präsenzformat. Im Telemedizinformat kann das Training Studierenden unabhängig von deren Standort angeboten werden.

## Introduction

Competence-based medical education is supposed to prepare medical students for clinical problem solving [1]. Focused history taking, knowledge-based clinical reasoning, and adequate case presentation represent important facets of competence which are crucial for good patient management [2], [3], [4]. To acquire these competences, communication trainings with simulated patients, seminars, and other training programs have been established in undergraduate medical education [5], [6], [7], [8]. Since the COVID-19 pandemic began, undergraduate medical education encountered a cease of classroom courses and bedside teaching [9]. In patient care, the number of telemedicine-based patient consultations increased enormously during the COVID-19 pandemic and will presumably continue at a high rate for much longer [10]. For medical education, the use of telemedicine has been proposed for the core entrustable professional activities (EPA) defined by the Association of American Medical Colleges (AAMC), including virtual history taking, documentation of clinical encounters, and giving or receiving a patient hand-off [11]. 

## Project description

In 2019, we developed a competence-based training for final-year medical students in the newly founded Center for Training and Assessment of Medical Competences at the University Hospital Hamburg Eppendorf based on a validated 360-degree assessment simulating a first day of residency [8]. This training included a consultation hour with four simulated patients per participant, patient documentation and management with a newly developed electronic patient chart, and one case presentation per participant in hand-off format. The patients designed for this training are shown in table 1 (Tab. 1). All patient cases were adapted from real patients who had been treated in the emergency department of the University Hospital Hamburg-Eppendorf. Results of the physical examination and a set of basic laboratory results were given to the participants after every simulated patient encounter. Before the case presentation, every participant received further results for one patient, e.g. ECG, X-rays or results of further blood tests. In October and December 2019 (presence), 103 medical students (63.1% female) participated in this training. At the end of each training, an electronic evaluation was presented to the participants. All consultations and case presentations were videographed. The simulated patients filled out a questionnaire (ComCare) including aspects of communication and interpersonal skills after every consultation [12]. Further trainings were planned for 2020. However, due to the social distancing regulations taking effect in March 2020, these trainings could not take place in their established way. Thanks to the funding of ten tablet computers we were able to offer the training via telemedicine. Via Zoom, virtual rooms were established for the consultations with the simulated patients, for working with the electronic patient charts, and for the case presentations. In May 2020 (telemedicine), 32 medical students (56.3% female), participated in the telemedicine-based training. This training was also electronically evaluated by the participants. All participants answered questions regarding the content of the training. Participants of the telemedicine-based training additionally answered questions with respect to the format of the training. All items were assessed on a 5-point Likert scale (1: does not apply, 2: somewhat applies, 3: partly applies, 4: rather applies, 5: fully applies). Comparisons with respect to the training content were calculated with t-tests for independent samples. Significance levels were set to p<0.05.

## Results

All 135 participants completed the training and no critical problems occurred in either format. Small technical problems during the telemedicine format were easily solved. All participants seemed to be very satisfied with the content of the training (see table 2 (Tab. 2)). No significant differences were found between the presence and the telemedicine group. Both groups regarded the patient cases as interesting (presence: 4.68±0.49, telemedicine: 4.66±0.48) and hardly knew the solution to the patient cases immediately (presence: 2.75±0.88, telemedicine: 2.75±0.76). With respect to the telemedicine format of the training (see table 3 (Tab. 3)), participants found it very useful (4.94±0.24) as a training for their final examination and felt that trainings like this should be offered throughout the final year (4.94±0.24). Participants felt that the telemedicine training was useful to exercise taking over responsibility as a physician (4.75±0.50) and that it should be offered in undergraduate medical education starting in year 4 (4.56±0.61).

## Discussion and conclusion

Changing a training that simulates a first day of residency from a presence to a telemedicine format led to no significant changes in the high evaluation of the usefulness of the training content. Participants wished to be offered more possibilities for training exercises in this format which might – with its design and content – help to improve the transition from undergraduate to postgraduate medical training [13]. Since students reported – independently of the training format – that they did not know the solutions to the patient cases immediately, our training seems to be a useful teaching tool to improve students’ diagnostic decision making [14]. The context of the patient cases, which is relevant for clinical reasoning [15], was designed in such a way, that both ways of clinical reasoning, pattern recognition and analytical thinking [16], had to be applied in patient workup. This seems to have worked successfully. In its telemedicine format, our competence-based training can be easily offered to final-year medical students independently of their current study location.

## Funding

This work was supported by the Joachim Herz Stiftung. The Claussen Simon Siftung provided additional support for five tablet computers.

## Ethical approval

The study was performed in accordance with the Declaration of Helsinki and the Ethics Committee of the Chamber of Physicians (Ethik-Kommission, Ärztekammer Hamburg), Hamburg, approved this study and confirmed its innocuousness. The study included written consent by the participants and participation was voluntary and anonymized (reference number: PV3649).

## Acknowledgements

We thank all participating medical students and the actors and actresses Christian Bruhn, Christiane Filla, Franziska Herrmann, Ulrike Johannson, Thomas Klees, Thorsten Neelmeyer, Frank Thomé, and Claudia Wiedemer who helped us to make this training work despite the COVID-19 pandemic.

## Competing interests

The authors declare that they have no competing interests. 

## Figures and Tables

**Table 1:**
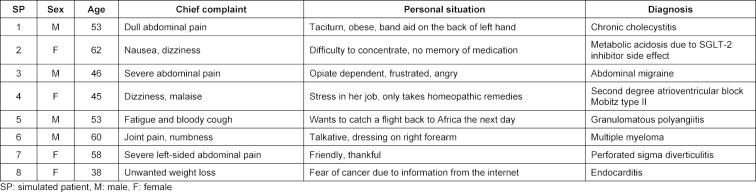
Simulated patient roles

**Table 2:**
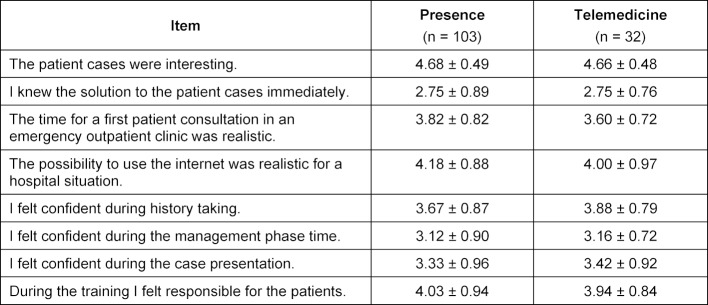
Students’ evaluation of the training content

**Table 3:**
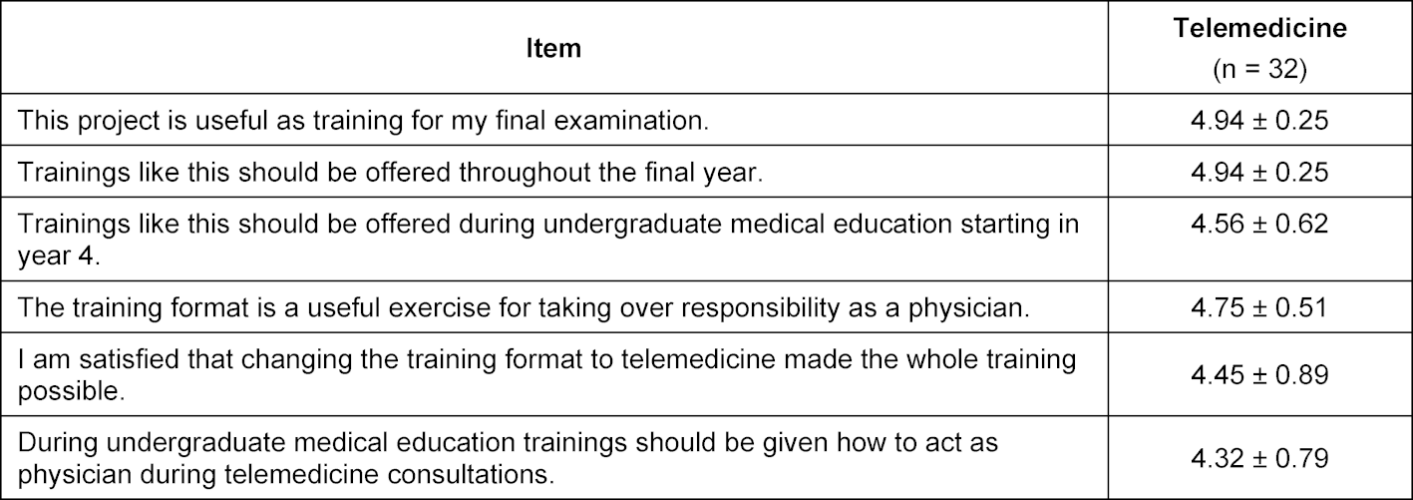
Students’ evaluation of the telemedicine training

## References

[1] Ten Cate O, Snell L, Carraccio C (2010). Medical competence: the interplay between individual ability and the health care environment. Med Teach.

[2] Dumont-Driscoll MC (2015). Foreword: too little, too late, too much, too long, just right? Reinforcing the importance of a thorough history and physical exam for correct diagnosis and ongoing patient management. Curr Probl Pediatr Adolesc Health Care.

[3] Melvin L, Cavalcanti RB (2016). The oral case presentation: a key tool for assessment and teaching in competency-based medical education. JAMA.

[4] Jain V, Rao S, Jinadani M (2019). Effectiveness of SNAPPS for improving clinical reasoning in postgraduates: randomized controlled trial. BMC Med Educ.

[5] Simmenroth-Nayda A, Weiss C, Fischer T, Himmel W (2012). Do communication training programs improve students' communication skills? - A follow-up study. BMC Res Notes.

[6] Harendza S, Krenz I, Klinge A, Wendt U, Janneck M (2017). Implementation of a clinical reasoning course in the Internal Medicine trimester of the final year of undergraduate medical training and its effect on student' case presentation and differential diagnostic skills. GMS J Med Educ.

[7] Royce CS, Atkins KM, Medniola M, Ricciotti H (2016). Teaching patient handoffs to medical students in obstetrics and gynecology: simulation curriculum and assessment tool. MedEdPORTAL.

[8] Prediger S, Schick K, Fincke F, Fürstenberg S, Oubaid V, Kadmon M, Berberat PO, Harendza S (2020). Validation of a competence-based assessment of medical students' performance in the physician's role. BMC Med Educ.

[9] Rose S (2020). Medical student education in the time of COVID-19. JAMA.

[10] Contreras CM, Metzger GA, Beane JD, Dedhia PH, Eiaz A, Pawlik TM (2020). Telemedicine: patient-provider clinical engagement during the COVID-19 pandemic and beyond. J Gastrointest Surg.

[11] Iancu AM, Kemp MT, Alam HB (2020). Un-muting medical student education: utilizing telemedicine during the COVID-19 pandemic and beyond. J Med Internet Res.

[12] Gärtner J, Prediger S, Harendza S (2020). Development and pilot test of ComCare – a questionnaire for quick assessment of communicative and social competences in medical students after interviews with simulated patients. GMS J Med Educ.

[13] Morgan HK, Meijcano GC, Skochelak S, Lmis K, Hawkins R, Tunkel AR, Nelson EA, Henderson D, Shelgikar AV, Santen SA (2020). A responsible educational handover: improving communication to improve learning. Acad Med.

[14] Prakash S, Sladek RM, Schuwirth L (2019). Interventions to improve diagnostic decision making: a systematic review and meta-analysis on reflective strategies. Med Teach.

[15] Daniel M, Durning SJ, Wilson E, Badoler E, Torre D (2020). Situated cognition: clinical reasoning and error are context dependent. Diagnosis (Berl).

[16] Monteiro SM, Norman G (2013). Diagnostic reasoning: where we've been, where we're going. Teach Learn Med.

